# Health-related quality of life up to 1 year after radiotherapy in patients with head and neck cancer (HNC)

**DOI:** 10.1186/s40064-016-2295-1

**Published:** 2016-05-20

**Authors:** Vera Loorents, Johan Rosell, Helen Salgado Willner, Sussanne Börjeson

**Affiliations:** Department of Radiation Oncology, Region Ostergotland, Linköping University Hospital, 58185 Linköping, Sweden; Regional Cancer Center South East Sweden and Department of Clinical and Experimental Medicine, Linköping University, Linköping, Sweden; Department of Medical and Health Sciences, Linköping University, Linköping, Sweden

**Keywords:** Health-related quality of life, Head and neck cancer, EORTC QLQ C30, QLQ-H&N35, Trismus

## Abstract

**Background:**

Detailed symptom specific descriptions of health-related quality of life (HRQOL), using validated questionnaires in patients with head and neck cancer (HNC) are sparse. The aim of the present study was to investigate HRQOL in patients with HNC up to 1 year after radiotherapy (RT), using two standardised questionnaires.

**Methods:**

The data for the present study was originally collected in a randomised, prospective study. Forty-seven patients from two RT clinics in Sweden were included to investigate the secondary aim: HRQOL. Data was recorded at baseline, completion of RT, and 3, 6, 12 months after completed RT, using the questionnaire EORTC QLQ-C30-version 3 and the disease-specific module EORTC QLQ-H&N35.

**Results:**

Most symptoms and functions deteriorated significantly by the end of RT, improved gradually by 3 and 6 months and reached baseline levels at 12 months after completed RT. However, 1 year after completed RT there were remaining significant problems in senses, dry mouth and sticky saliva.

**Conclusions:**

Radiation therapy affects health-related quality of life in patients with head and neck cancer, both in the short and long term. Caregivers need management strategies for early detection and treatment of specific problems throughout the treatment period to help in the prevention of long-term symptoms.

**Electronic supplementary material:**

The online version of this article (doi:10.1186/s40064-016-2295-1) contains supplementary material, which is available to authorized users.

## Background

In Sweden, approximately 1200 individuals are diagnosed with HNC every year. Standard treatments for patients with HNC include RT, with or without surgery, and/or combined chemotherapy (CT) (Socialstyrelsen 2011). Advancements in RT treatment techniques in patients with HNC have resulted in better survival rates (Beadle et al. 2014) but have also induced challenges related to management of acute and late side effects of RT. Some of these side effects can be objectively observed, but many of them can only be measured by the patients themselves. Quality of life questionnaires measuring symptoms of the disease and side effects of treatment gives the patient a structured tool for expression and provides health caregivers with valuable information on patient reported outcomes. This ultimately enhances the understanding of patient burden in the development of new treatment techniques.

Health-related quality of life (HRQOL) is defined as a specific subset of quality of life, assessing symptoms, psychological aspects, and function (Fayers and Machin 2007). Overall HRQOL in patients with HNC has been assessed in several studies using the validated EORTC QLQ-C30 and QLQ-H&N35 instruments (Aaronson et al. 1993; Bjordal et al. 1999). Results from previous studies on HRQOL have shown that most of the patient’s function, symptom and global health scales show deterioration at the completion of RT and then an improvement by 3 and 6 months after completed RT (Chandu et al. 2006; Murphy et al. 2007; Sherman and Simonton 2010; So et al. 2012). Some symptoms including xerostomia, changes in taste and smell, sticky saliva and deterioration in physical functioning are still remaining up to 12 months after completed RT (So et al. 2012). A review reporting on patient-reported late side effects of RT from 12 months to 5 years after completed treatment showed worsening of role functioning, sticky saliva, nausea and trismus (Sherman and Simonton 2010). Despite the fact that studies in the past 20 years have been reporting on aspects of HRQOL among patients with HNC (Bjordal et al. 1994, 2001; de Graeff et al. 2000; Rathod et al. 2013), few studies have monitored patients with close follow-ups over time from the completion of RT up to 1 year after RT, using all the HRQOL aspects outlined in the EORTC QLQ-C30 and QLQ-H&N35. Detailed follow-up is important if health care providers are to succeed in supporting specific needs at specific time points in the patient recovery.

The data for the present study was collected in a randomised, prospective study investigating the effectiveness of a training intervention to prevent trismus in patients undergoing RT and up to 12 months after completed RT (primary endpoint). No statistically significant differences in trismus were found between the intervention and control groups (Loorents et al. 2014).

Thus, the aim of the present study was to investigate HRQOL in patients with HNC over time and up to 1 year after RT, using two standardised questionnaires.

## Methods

### Study design and participants

This is a longitudinal descriptive study including patients with HNC receiving RT. Head and neck cancer is defined as a tumour arising from the mucosal surfaces of the lip, oral cavity, pharynx, larynx or cervical oesophagus. Other sites included are the nose/paranasal sinuses, and salivary/thyroid/parathyroid glands (MeSH Browser 1997). Sixty-six consecutive patients from two different RT clinics in Sweden were initially included between 2009 and 2013. Out of these, 47 completed the 12-month follow-up time period and were therefore included in the present study.

### Inclusion criteria

Patients were included if they were willing to participate, >18 years, and able to understand and communicate in Swedish. Other study criteria are outlined in the report from the main study (Loorents et al. 2014).

### Study procedures

#### Radiotherapy and treatments

All 47 patients received external beam radiotherapy (RT). Treatment regimens were given in accordance with tumour size, site and stage of disease. Radiotherapy was administered in either curative or palliative intentions, pre or post-surgery. Therapy doses ranged between 46 and 70 Gy given in 2 Gy daily, 5 days a week. Treatment techniques included 3D-CRT or IMRT.

#### Data collection

Demographic variables, listed in Table 1, were collected in a written questionnaire, while clinical data was extracted from patient medical records.

**Table 1:** Patient characteristics (n = 47)

Variables	(n = 47)
Gender	
Male	39
Female	8
Age (mean, SD, range)	63.13, 11.3, 24–88
Tumour site	
Base of tongue	8
Tongue	2
Nasal cavity	1
Floor of mouth	2
Parotid gland	10
Tonsil	19
Oropharynx/hypoparynx	1
Larynx	1
Lymph, secondary tumour	3
Tumour size	
Tl	8
T2	20
T3	5
T4	5
Missing data	9
External beam radiotherapy (RT)	
3D conformal radiotherapy (3DCRT)	24
Intensity-modulated radiotherapy (1MRT)	23
Concomitant chemotherapy^a^	27
No chemotherapy	20

^a^Chemotherapy: Cisplatin 50 mg once a week

#### Health-related quality of life assessments

Health-related quality of life was assessed at five different points; baseline, at completion of RT, 3, 6, and 12 months after completed RT, using the self-administered EORTC QLQ-C30-version 3 and the disease specific module EORTC QLQ-H&N35. The baseline measurement was assessed the week prior to RT start. Patients received their first two questionnaires from their contact nurse. After completion of RT, the questionnaires were sent by post together with a pre-paid return envelope addressed to one of two assessors. A reminder questionnaire was sent to non-responders. A total of 330 questionnaires plus reminder questionnaires were distributed. Dates for distribution of the questionnaires were calculated from the date for the baseline questionnaire.

The European Organisation for the Research and Treatment of Cancer’s Quality of Life Questionnaire, Core-30-version 3 (EORTC QLQ-C30), and specific module Head and Neck 35 (EORTC QLQ-H&N35) are proven to be robust instruments and have a widespread clinical usage. The core instrument EORTC QLQ-C30 is used in conjunction with site or disease modules to provide a more comprehensive assessment of patient reported difficulties. Established reliability, validity and sensitivity to change have been tested in many clinical studies (Aaronson et al. 1993; Rathod et al. 2013).

In both questionnaires a 1-week time frame is used. Most questions are answered on a scale from 1 to 4 where 1 = not at all, 2 = a little, 3 = quite a bit, and 4 = very much. Global health status uses numerical scales (1–7), where 1 = very poor and 7 = excellent. Some symptom questions in the H&N35 module are answered by yes or no.

Health-related quality of life scores are then calculated according to the EORTC QLQ-C30 scoring manual *EORTC QLQ*-*C30 (version 3)* (Fayers et al. 2001). All of the EORTC scales and single-item score measures are converted to a scale ranging from 0 to 100, except the global health scales, which range from 1 to 7.

#### EORTC QLQ-C30

This core questionnaire includes 30 items, that describe 5 functioning scales (physical, role, emotional, cognitive, and social functioning), 3 symptom scales (fatigue, nausea/vomiting, and pain), and 6 single items symptoms (dyspnoea, insomnia, loss of appetite, constipation, diarrhoea, and financial difficulties). There is also a global health status scale.

Higher scores on the 5 functional and global health status scales refer to better health status, while higher scores on the symptom scales represent more problems.

#### EORTC QLQ-H&N35

This module was developed specifically for patients with HNC and contains 35 questions divided into 7 subscales about pain, swallowing, senses, speech, social eating, social contact, and sexuality. There are 10 single items relating to problems with teeth, dry mouth, cough, mouth opening, sticky saliva, weight loss, and weight gain, use of nutritional supplements, feeding tubes, and painkillers. Higher scores in this module represent higher level of problems.

#### Sample size calculation and statistical analysis

The sample size was calculated from the main outcome reported elsewhere (Loorents et al. 2014). Of the original 66 patients, 47 fulfilled the 12-month assessment and were therefore included in the present analysis on HRQOL. The 19 dropouts were a result of death (n = 5), failure to comply (n = 7), logistical problems (n = 1), or withdrawal due to patient request (n = 6) (Loorents et al. 2014). All data was entered into an Access database, and all statistical analyses were done with IBM SPSS-(version 21).

To investigate the differences between study groups, the Mann-Whitney U test was used.

For pairwise comparisons the Wilcoxon signed-rank test was conducted. All *p* values were two-sided, and results were considered significant at *p* < 0.05.

## Results

A total of 47 patients completed the 12-month follow-up. To investigate if we could analyse the data on HRQOL for the entire group of patients with HNC, irrespective of the original study groups we preformed comprehensive analysis on differences in HRQOL between the intervention/control groups from the original study (Loorents et al. 2014). No significant differences in any of the functional or global HRQOL scales were found between the groups However, there were statically significant differences in a few of the symptom scales, including:

The control group used more nutritional supplements (mean = 63) than the intervention group (mean = 33) at baseline (p = 0.018).

The control group self-reported more difficulty in opening their mouth (mean = 24) than the intervention group (mean = 15) at the completion of RT (p = 0.0039). This difference was also found at 3 months after completed RT, where the control group still had difficulty in opening their mouth (mean = 17) compared to the intervention group (mean = 9), (p = 0.031). The intervention group had more problems (mean = 5) than the control group (mean = 0) with nausea/vomiting at 12 months after completed RT (p = 0.017).

Due to the homogeneity of the two original study groups, we decided to analyse the results regarding quality of life in the entire group of head and neck cancer patients, addressing all assessment points, regardless of original group affiliation.

Baseline demographic variables for this present study group are outlined in Table 1.

### Changes over time in HRQOL

**Fig. 1:**
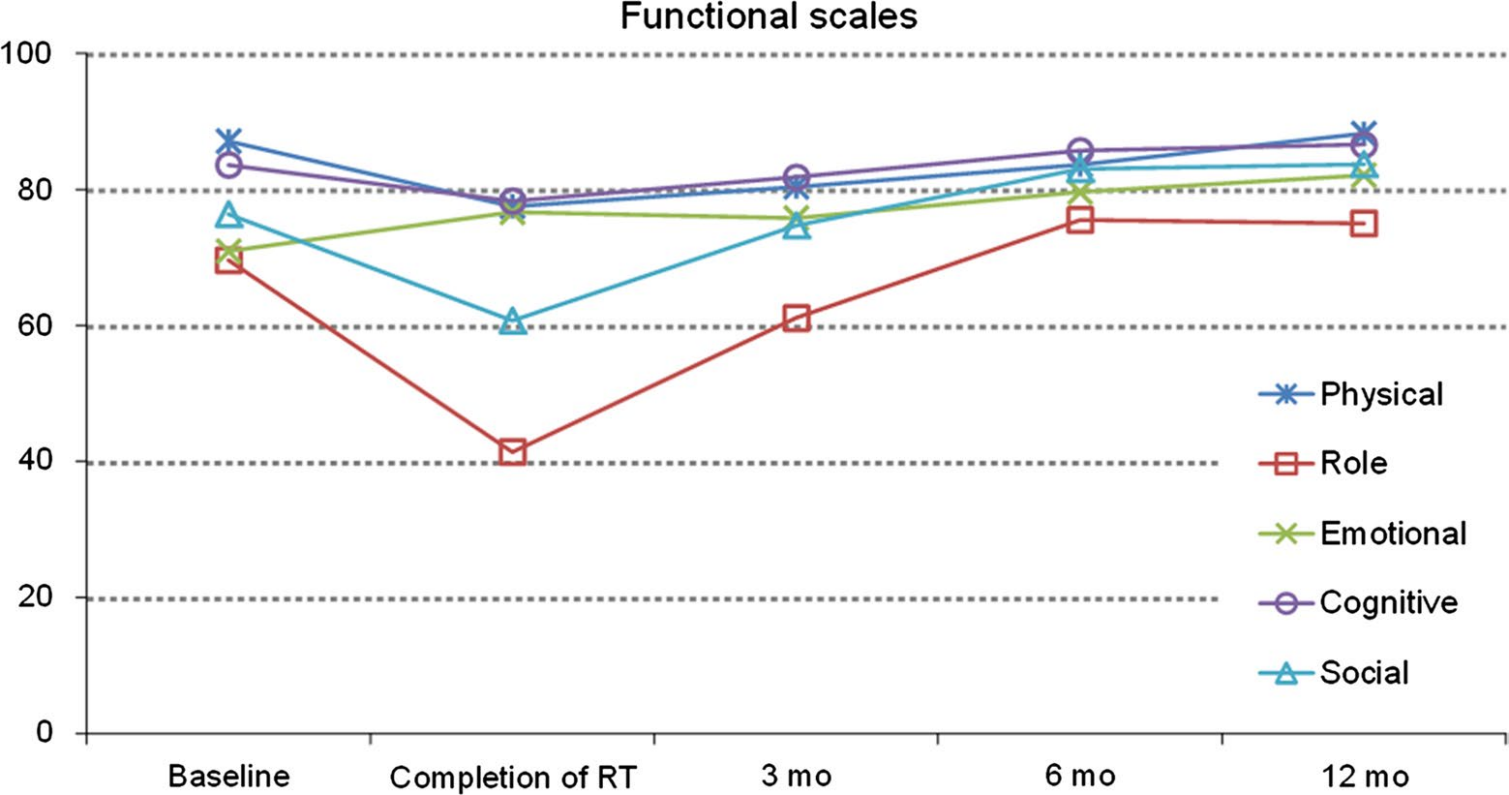
Functional scales of the EORTC QLQ C-30 (0–100 point scale) from baseline over a 12-month period. Mean values based on patients answering the questionnaire. Higher scores indicate a high level of function. Statistically significant differences compared to baseline: <0.001 at completion of RT in physical, role, and social scales; <0.05 at completion of RT in cognitive scale; <0.001 at 3 months after RT in physical scale; <0.05 at 3 months in role scale; <0.001 at 6 months in physical scale; <0.05 at 6 months in emotional scale; <0.05 at 12 months in emotional scale

There was a significant deterioration from baseline to completion of RT in most of the functioning scales, resulting in more problems in the physical, role, cognitive and social scales (Fig. 1).

There was still a significant worsening from baseline to the 3-month follow-up in some of the functioning scales, resulting in problems in, the physical and role functioning scales (Fig. 1). This remained at 6 months after completed RT in the physical and functioning scales.

At 12 months, all of the scales had gradually increased to above baseline values.

**Fig. 2:**
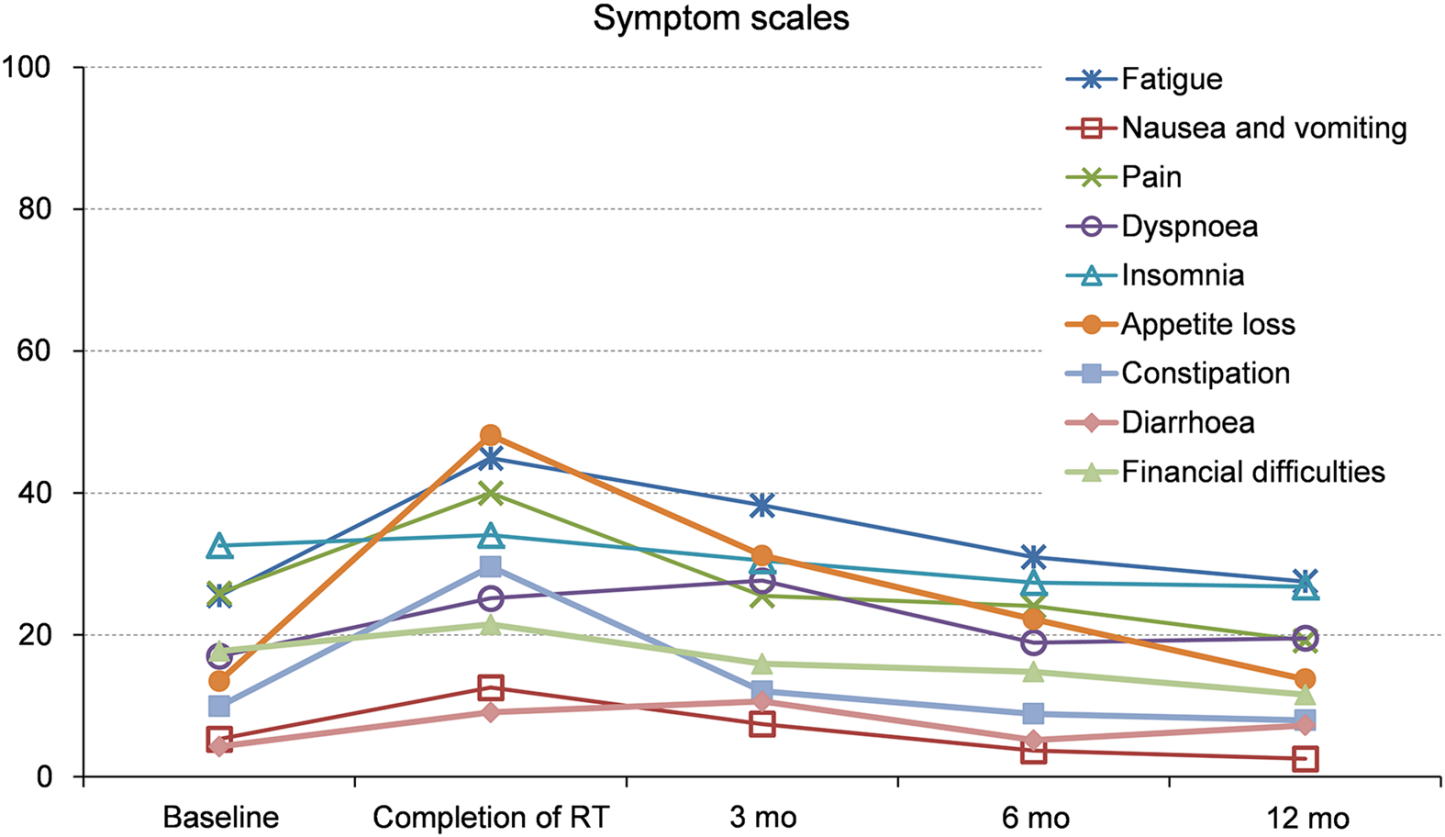
Symptom scales of the EORTC QLQ C-30 (0–100-point scale) from baseline over a 12-month period. Mean values based on patients answering the questionnaire. Higher scores indicate more severe symptoms or impairments. Statistically significant differences compared to baseline: <0.001 at completion of RT in fatigue, appetite loss, and constipation; <0.05 at completion of RT in nausea, pain, and dyspnoea; <0.001 at 3 months after RT in fatigue, and appetite loss; <0.05 at 3 months in dyspnoea, and diarrhoea; <0.05 at 6 months in fatigue, and appetite

**Fig. 3:**
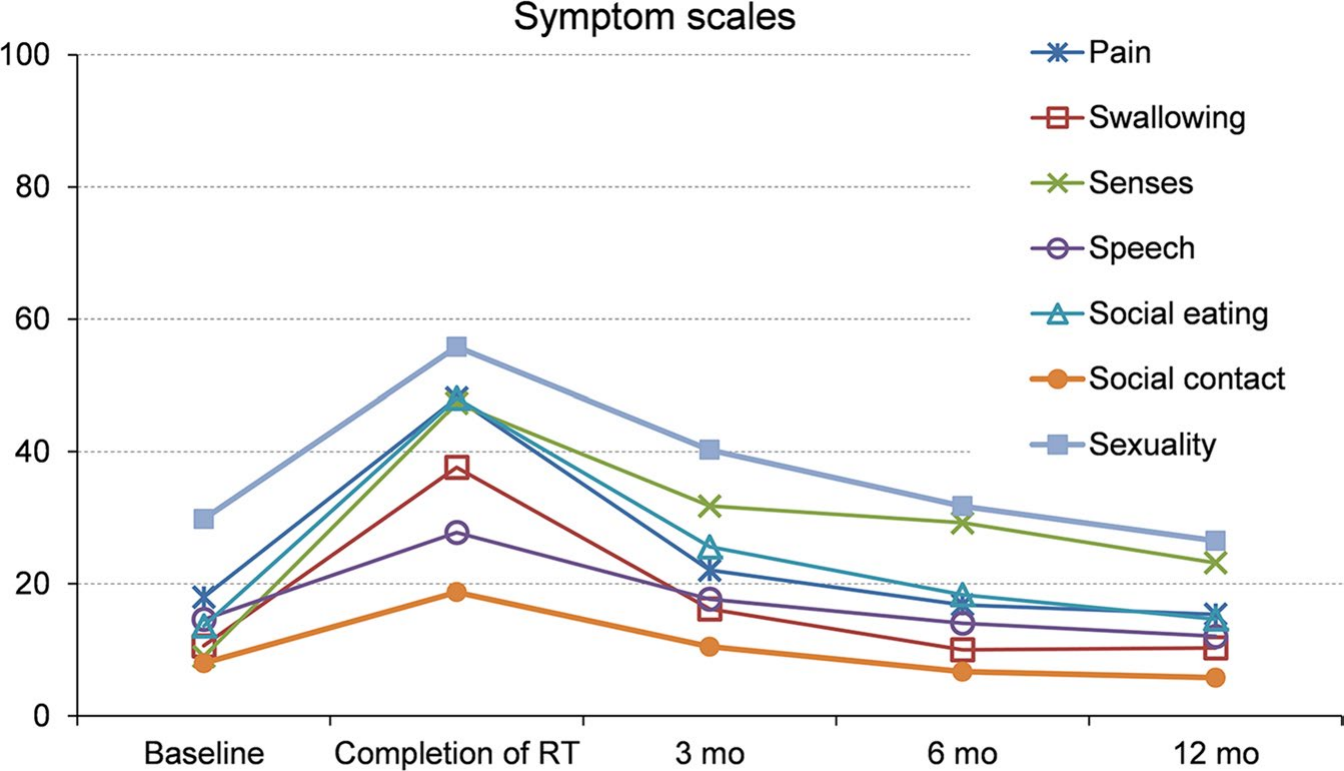
Symptom scales of the EORTC QLQ-H&N35 (0–100-point scale) from baseline over a 12-month period. Mean values based on patients answering the questionnaire. Higher scores indicate more severe symptoms or impairments. Statistically significant differences compared to baseline: <0.001 at completion of RT in pain, swallowing, senses, social eating, and sexuality; <0.05 at completion of RT in social contact, and speech; <0.001 at 3 months after RT in senses, and social eating; <0.05 at 3 months in sexuality; <0.001 at 6 months in senses; <0.05 at 6 months in social eating; <0.001 at 12 months in senses

**Fig. 4:**
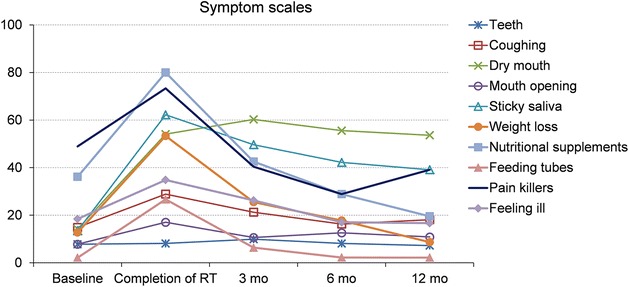
Symptom scales of the EORTC QLQ-H&N35 (0–100-point scale) from baseline over a 12-month period. Mean values based on patients answering the questionnaire. Higher scores indicate more severe symptoms or impairments. Statistically significant differences compared to baseline: <0.001 at completion of RT in dry mouth, sticky saliva, weight loss, and nutritional supplements; <0.05 at completion of RT in feeling ill, pain killers, feeding tubes, mouth opening, and coughing; <0.001 at 3 months after RT in dry mouth, and sticky saliva; <0.001 at 6 months in dry mouth, and sticky saliva; <0.05 at 6 months in pain killers; <0.001 at 12 months in dry mouth, and sticky saliva

There was a significant decrease in global health scales at the completion of RT compared to baseline. There was a significant improvement in global health scales compared to baseline at 3, 6 and 12 months after completed RT, resulting in better values than baseline global health values (Additional file 1). Some symptoms, single items and subscales were significantly increased in both the EORTC QLQ-C30-version 3 and EORTC QLQ-H&N35 at the completion of RT compared to baseline, resulting in problems in: fatigue, nausea/vomiting, pain, dyspnoea, appetite loss, constipation, mouth opening, dry mouth, sticky saliva, coughing, pain killers, nutritional supplements, feeding tubes, weight loss, swallowing, senses, speech, social eating, social contact, less sexuality, feeling ill (Figs. 2, 3, 4).

A few of the above items remained a significant problem at 3 months after completed RT compared to baseline, namely fatigue, appetite loss, senses, social eating, less sexuality, dry mouth, sticky saliva, and dyspnoea (Figs. 2, 3, 4).

At 6 months there were still remaining problems in fatigue, appetite loss, senses, social eating, dry mouth, sticky saliva, and pain killers compared to baseline (Figs. 3, 4).

Remaining significant increases at 12 months were found in senses, dry mouth, and sticky saliva, resulting in continued problems compared to baseline (Figs. 3, 4). Diarrhoea was significantly increased at 3 months after completed RT compared to baseline. At 6 months there were better values than at baseline, values had normalised at 12 months (Fig. 2).

## Discussion

The results of this study show an overall decrease in HRQOL at the completion of RT and fast recovery during the follow-up year. Several symptoms and functions deteriorated significantly by the end of RT, and then gradually improved by 3 and 6 months to reach baseline levels at 12 months after completed RT.

However, at 1 year after completed RT there were remaining significant problems in senses, dry mouth and sticky saliva.

We chose to analyse the patients over time, irrespective of the original study group, as there were only small statistically significant differences between the original intervention and control groups. We were surprised about the significant differences in self-reported mouth opening between the two groups as we in the main study (Loorents et al. 2014) did not find significant differences in the same patients using standardised, objective measurements. Although the differences were statistically significant they were small and of little clinical relevance, which supports our conclusion in the main study that mouth opening training cannot be recommended for all patients receiving RT.

The present study has a more positive outcome on global health over time than other studies that have reported on a more gradual increase in global health scales over time from baseline and after completed RT (Murphy et al. 2007; So et al. 2012). The slight decrease in global health values observed at the completion of RT in this study may have been the result of a higher usage of feeding tubes at this measuring point. One review (Murphy et al. 2007) suggested that feeding tubes are the single most powerful predictor for worsened QoL after RT. Overall global health improvement may also be explained by patients’ adjustment to a new life situation and life appraisal. We assume that a patient who has learnt to cope adjusts better to their situation. Sherman and Simonton (2010), reported on how patients cope with HNC, suggesting that patients undergoing treatments use the greatest number of coping strategies. Hammerlid and Taft (2001), reporting on a comparison between HNC patients and the general Swedish population with regard to HRQOL 3 years after treatments, showed that patients with HNC still suffer functional problems related to their disease/treatments. However, these problems do not generally affect their overall HRQOL, which suggests that patient have successfully adjusted to living with their problems.

Patients' grading of the function scales were high at baseline. The largest decrease was reported in role and social functioning, which was only fully restored at the 6-month assessment. All function scales were above baseline values at 12 months. Some studies have reported on a decrease in physical functioning remaining at 12 months and up to 5 years (Sherman and Simonton 2010; So et al. 2012), while Murphy et al. (2007) reported on QoL declining immediately after therapy (RT + surgery) and returning towards baseline by 1 year (Additional file 1).

Emotional role was scored higher than expected at baseline (indicating fewer problems) in the present study, reaching significantly better values than baseline levels at 6 months. This low incidence of emotional role problems may have to do with the fact that there were more men included in the study; one study reported that women have more problems with emotional role (Hammerlid et al. 2001). A review from 2010 (So et al. 2012) reported an emotional function scoring deterioration during RT and a stabilisation at 12 months. Our patients were all offered professional counselling by hospital counsellors, as part of our hospitals standard care at the time of their cancer diagnosis. This may be a contributing factor to the positive emotional function scores in the present study.

Cure and tumour control has been the focus of HNC management, with less focus on quality of life and rehabilitation. In daily clinical work, improvements in quality assessments of oral function during and after treatments are needed to help manage oral function and prevent chronic sequelae. The remaining chronic problems in senses, dry mouth and sticky saliva observed in the present study have been reported in other studies (Bjordal et al. 1994, 2001). One review reported that the best results for reducing acute oral toxicities of cancer therapies are achieved with a multidisciplinary health care team who communicate effectively to coordinate patient care (Epstein et al. 2012). Good communication and access to HRQOL data may play a role in QoL issues for HNC patients. There is indication of a positive therapeutic effect on HRQOL in HNC patients who experience that their physicians have access to OoL data and use this information in their communication (Murphy et al. 2007).

Health care providers need to address what types of interventions are needed and when they are needed in the supportive care for these patients. Are men and women’s needs different? What do patients perceive as problems? For example a recent review reported poorer QoL in women (Sherman and Simonton 2010). In our study only eight women participated making it impossible to do further analysis regarding gender differences, but it needs to be addressed in future studies. Perceptions of complications after RT was investigated among 33 patients after RT, showing that lethargy, weakness, dry mouth, mouth sores, pain, taste changes, and sore throat were the most debilitating side effects, particularly oropharyngeal mucositis (Rose-Ped et al. 2002).

### Study strengths

The main strength of the present study is the complete in-depth presentation of the whole quality of life tool. There is data for all 47 patients who completed all the measurements, which provides a stable analysis of changes over time.

### Study limitations

Even though prospective data was used in the data collection, the original study design was to test two groups. This needs to be taken into account when interpreting the results. There may not be enough power in the analysis to support the secondary aim of this study group, although this may be justified by the significant number of differences found over time. This was a selected group as some patients were excluded from participation in the main intervention study. For example, no edentulous patients were included.

## Conclusion and clinical implications

The present study highlights the different problem areas in HRQOL over time, both in the short and long term, in patients with head and neck cancer. This is important information when planning the time for implementation of an intervention in supportive care. Caregivers need management strategies for specific problems throughout the treatment period to help with the prevention of long-term symptoms. Caregivers need management strategies for early detection and treatment of specific problems throughout the treatment period to help in the prevention of long-term symptoms.

## Future studies

Future prospective studies are needed to identify factors associated with more impaired HRQOL and effective interventions against the problem areas highlighted by patients. Furthermore, there is a need to compare similarities and differences in patient-reported outcomes, such as assessment of mouth opening with standardised measurements.

## Additional file

10.1186/s40064-016-2295-1 Global health – quality of life as measured with EORTC QLQ C-30 (0-100- point scale) from baseline over a 12 month period. Mean values based on patients answering the questionnaire. Higher scores indicate a high level of quality of life.

## Abbreviations

HNC: head and neck cancer
HRQOL: health-related quality of life
RT: radiotherapy
EORTC: European Organisation for the Research and Treatment of Cancer
QLQ: Quality of Life Questionnaires
H&N: Head and Neck
CT: chemotherapy
