# Supplementary material for: Health-related quality of life up to 1 year after radiotherapy in patients with head and neck cancer (HNC)
Source: Springerplus. 2016 May 20;5(1):669. doi: 10.1186/s40064-016-2295-1 (PMC4899339; doi:10.1186/s40064-016-2295-1)
Supplement: Supplementary file 1 — 10.1186/s40064-016-2295-1 Global health – quality of life as measured with EORTC QLQ C-30 (0-100- point scale) from baseline over a 12 month period. Mean values based on patients answering the questionnaire. Higher scores indicate a high level of quality of life. [file 40064_2016_2295_MOESM1_ESM.docx]

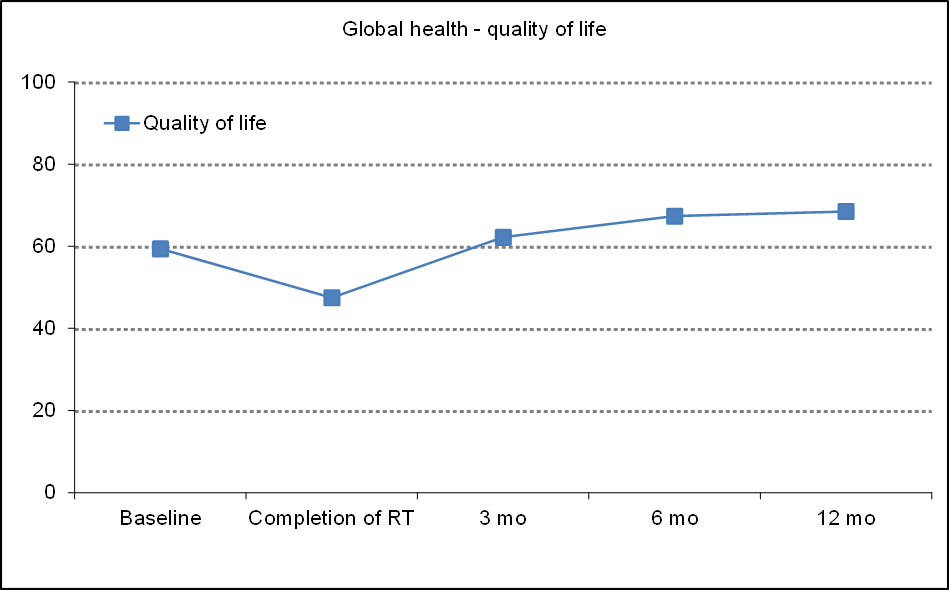


Global health – quality of life as measured with EORTC QLQ C-30 (0-100- point scale) from baseline over a 12 month period. Mean values based on patients answering the questionnaire. Higher scores indicate a high level of quality of life.
